# Mechanistic insights into endosomal escape by sodium oleate-modified liposomes

**DOI:** 10.3762/bjnano.15.131

**Published:** 2024-12-30

**Authors:** Ebrahim Sadaqa, Satrialdi, Fransiska Kurniawan, Diky Mudhakir

**Affiliations:** 1 Department of Pharmaceutics, School of Pharmacy, Institut Teknologi Bandung (ITB), Bandung 40132, Indonesia; 2 Department of Pharmacochemistry School of Pharmacy, Institut Teknologi Bandung (ITB), Bandung 40132, Indonesia

**Keywords:** Aurein 1.2, endosomal escape, fusogenic effect, molecular dynamics simulation, sodium oleate

## Abstract

Endosomal entrapment significantly limits the efficacy of drug delivery systems. This study investigates sodium oleate-modified liposomes (SO-Lipo) as an innovative strategy to enhance endosomal escape and improve cytosolic delivery in 4T1 triple-negative breast cancer cells. We aimed to elucidate the mechanistic role of sodium oleate in promoting endosomal escape and compared the performance of SO-Lipo with unmodified liposomes (Unmodified-Lipo) and Aurein 1.2-modified liposomes (AUR-Lipo). Liposomes were prepared using the thin-film hydration method, resulting in Unmodified-Lipo, SO-Lipo, and AUR-Lipo formulations. The particle sizes were 102.2 ± 3.30 nm for Unmodified-Lipo, 109.6 ± 7.65 nm for SO-Lipo, and 151.9 ± 5.88 nm for AUR-Lipo, with polydispersity indices below 0.25, indicating uniform size distribution. Endosomal escape efficiency was evaluated through confocal microscopy by measuring the colocalization of labeled liposomes with lysosomal markers, quantified using Pearson’s correlation coefficient. Lipid mixing assays assessed the potential fusogenic effect, and molecular dynamics (MD) simulations explored the interactions of protonated sodium oleate (SO) with the endosomal membrane. Results demonstrated that SO-Lipo exhibited superior endosomal escape compared to Unmodified-Lipo, as evidenced by reduced colocalization with lysosomal markers, and achieved comparable efficacy to AUR-Lipo with lower cytotoxicity. Lipid mixing assays confirmed the potential fusogenic effect of SO with endosomal membrane models. MD simulations revealed that under acidic endosomal conditions, SO is protonated to oleic acid, which integrates into the membrane, enhancing fluidity and promoting fusion events essential for cytosolic release. SO-Lipo enhance endosomal escape through a fusogenic mechanism, facilitating cytosolic delivery with reduced cytotoxicity. This approach offers a safer and more effective option for targeted drug delivery applications.

## Introduction

The quest for efficient drug delivery has spurred extensive research into liposomal systems. These vesicles, with their inherent ability to encapsulate and protect a diverse range of therapeutic agents, including small molecule drugs, proteins, and genetic material such as DNA and RNA, hold significant promise for transforming drug delivery methodologies [1]. Despite their potential, liposomes encounter substantial challenges from the point of administration to achieving therapeutic efficacy. One of the primary obstacles is their propensity for endosomal entrapment. Following internalization via endocytosis, liposomes are frequently confined within endosomes, where they risk degradation in lysosomes or expulsion back to the cell surface. This endosomal barrier critically impedes the effective release of encapsulated drugs into the cytosol, limiting their therapeutic impact [2–3]. Consequently, facilitating endosomal escape has emerged as a crucial step in the intracellular trafficking process, essential for achieving successful cytosolic delivery and realizing the full therapeutic potential of liposomal systems [4].

To address the formidable challenge of endosomal entrapment, several strategies have been developed. Cell-penetrating peptides (CPPs), renowned for their ability to traverse biological membranes, have been extensively studied for their potential to enhance endosomal escape by causing membrane disruption [5]. However, the broad utility of CPPs is limited by their non-specific nature, which often leads to toxicity and diminishes their therapeutic value [4,6]. Additionally, the necessity for chemical conjugation between CPPs and therapeutic agents introduces complexities that can affect the pharmacokinetic profile and biodistribution of the drug. Alternative approaches, such as the use of ionizable lipids or pH-sensitive polymers, have also been investigated. While promising, these methods face significant hurdles, including toxicity, instability under physiological conditions, reduced drug loading capacity, and complex synthesis processes that hinder widespread adoption [7–8].

Given the limitations of current endosomal escape strategies, innovative approaches are urgently needed. Unraveling the mechanisms underpinning endosomal escape is pivotal for the development of novel, safe, and effective agents capable of overcoming these formidable barriers. Recent studies have highlighted the potential of oleic acid (OLA), the protonated form of sodium oleate (SO), to enhance membrane fluidity in specific lipid bilayer models, including dioleoylphosphatidylcholine (DOPC) and dipalmitoylphosphatidylcholine (DPPC), as well as in complex biological systems such as the human skin barrier [9–11]. OLA, a monounsaturated fatty acid, possesses unique physicochemical properties that make it a promising candidate for facilitating endosomal escape. Its kinked hydrocarbon chain, featuring a double bond, disrupts lipid bilayers and increases membrane fluidity.

Inspired by these properties, we propose using anionic SO, a pH-sensitive lipid-based molecule with amphipathic characteristics, as an innovative agent for overcoming endosomal entrapment. At physiological pH, SO carries a negative charge, limiting its interaction with cell membranes. However, within the acidic endosomal environment, SO converts to its protonated form, OLA, enhancing its hydrophobic interactions with the endosomal membrane. This protonation is expected to further increase membrane fluidity, facilitating the release of the liposomal payload into the cytosol. Moreover, its amphipathic nature allows it to form stable complexes with liposomal components, enhancing both liposome stability and drug encapsulation.

To gain deeper insight into the mechanisms underpinning SO’s potential as an endosomal escape agent, molecular dynamics (MD) simulations have emerged as an indispensable computational tool. These simulations enable the exploration of complex lipid–membrane interactions and dynamic molecular processes at an atomistic level, offering unparalleled insights into mechanisms that are challenging to observe experimentally. By capturing processes such as membrane destabilization, pore formation, and lipid fusion, MD simulations bridge the gap between experimental observations and mechanistic understanding. They also provide a robust framework for designing and optimizing innovative strategies for endosomal escape. Understanding these molecular interactions is akin to solving a complex puzzle, where each detail contributes to a comprehensive picture of overcoming endosomal entrapment.

This study aims to bridge the knowledge gap by investigating the potential of SO in enhancing endosomal escape and elucidating the underlying mechanisms. We employ a multifaceted approach, combining in vitro experimentation, using 4T1 triple-negative breast cancer cell line as our in vitro model, with MD simulations to explore the intricate interactions between SO and the endosomal membrane. This allows us to elucidate the underlying mechanisms by which SO promotes endosomal escape. Additionally, we directly compare the endosomal escape efficacy of SO with Aurein 1.2 (AUR), a well-established endosomal escape peptide known for its efficacy both in vitro and in vivo, serving as a positive control [12].

Our findings have the potential to significantly advance the development of safer and more effective liposomal drug delivery systems. By presenting SO as a promising alternative endosomal escape agent, we aim to contribute to the advancement of targeted drug delivery strategies with improved therapeutic outcomes.

## Results and Discussion

### Characterization of liposomal formulations

In our study, we meticulously formulated three distinct liposomal variants to evaluate key nanocarrier quality attributes, including particle size, polydispersity index (PDI), and zeta potential. These assessments were conducted at both physiological pH (7.4) and acidic pH (5), as summarized in Table 1.

**Table 1:** Physicochemical properties of liposomal formulations at physiological and acidic pH (*n* = 3).

Sample	Mean particle size (nm) at pH 7.4	PDI at pH 7.4	Zeta potential (mV) at pH 7.4	Mean particle size (nm) at pH 5	PDI at pH 5	Zeta potential (mV) at pH 5

Unmodifed-Lipo	102.2 ± 3.30	0.239 ± 0.046	−4.47 ± 2.34	107.8 ± 3.40	0.201 ± 0.026	−5.57 ± 0.271
SO-Lipo	109.6 ± 7.65	0.233 ± 0.032	−24.12 ± 5.75	119.2 ± 2.54	0.257 ± 0.045	−0.86 ± 5.14
AUR-Lipo	151.9 ± 5.88	0.248 ± 0.040	−2.42 ± 2.41	149.6 ± 7.84	0.254 ± 0.019	1.07 ± 1.80

At physiological pH (7.4), the unmodified liposomes (Unmodified-Lipo) exhibited a stable physicochemical profile, with an average particle size of 102.2 ± 3.30 nm. Their PDI of 0.239 ± 0.046 indicated a uniform and consistent size distribution, supported by a mean negative zeta potential of −4.47 ± 2.34 mV. For sodium oleate-modified liposomes (SO-Lipo), a slight increase in mean particle size to 109.6 ± 7.65 nm was observed, which remained well within the preferred size range for nanoformulations. The PDI, averaging to 0.233 ± 0.032, was similar to that of the Unmodified-Lipo, suggesting that the incorporation of SO did not disrupt vesicle uniformity. However, the zeta potential significantly decreased to −24.12 ± 5.75 mV, reflecting a substantial change in surface charge due to the anionic nature of SO, which may enhance colloidal stability through electrostatic repulsion. The Aurein 1.2-modified liposomes (AUR-Lipo) showed a notable increase in particle size to 151.9 ± 5.88 nm, approaching the higher end of the optimal range for nanocarriers, while maintaining a PDI similar to Unmodified-Lipo at approximately 0.248 ± 0.040. Notably, the PDI values below 0.5 across all formulations suggested a high degree of homogeneity in our nanoformulations. Interestingly, the zeta potential of AUR-Lipo remained virtually unchanged at −2.42 ± 2.41 mV, indicating that the neutral charge of the AUR peptide effectively preserved the nanoparticle’s surface charge. When exposed to pH 5 for 1 h, Unmodified-Lipo maintained its size and charge, demonstrating notable stability across the studied pH range. In contrast, SO-Lipo exhibited a significant reduction in the negativity of its zeta potential (−0.86 ± 5.14 mV), consistent with the expected protonation of SO to OLA under acidic conditions. Most strikingly, AUR-Lipo underwent a critical transition, as evidenced by a shift in zeta potential to a slightly positive value of 1.07 ± 1.80 mV. This shift may be attributed to the protonation of amino acids such as glutamic and aspartic acid in the peptide sequence, which, under acidic conditions, likely contributes to an increase in positive charge [13].

### Cytotoxicity evaluation

**Figure 1:**
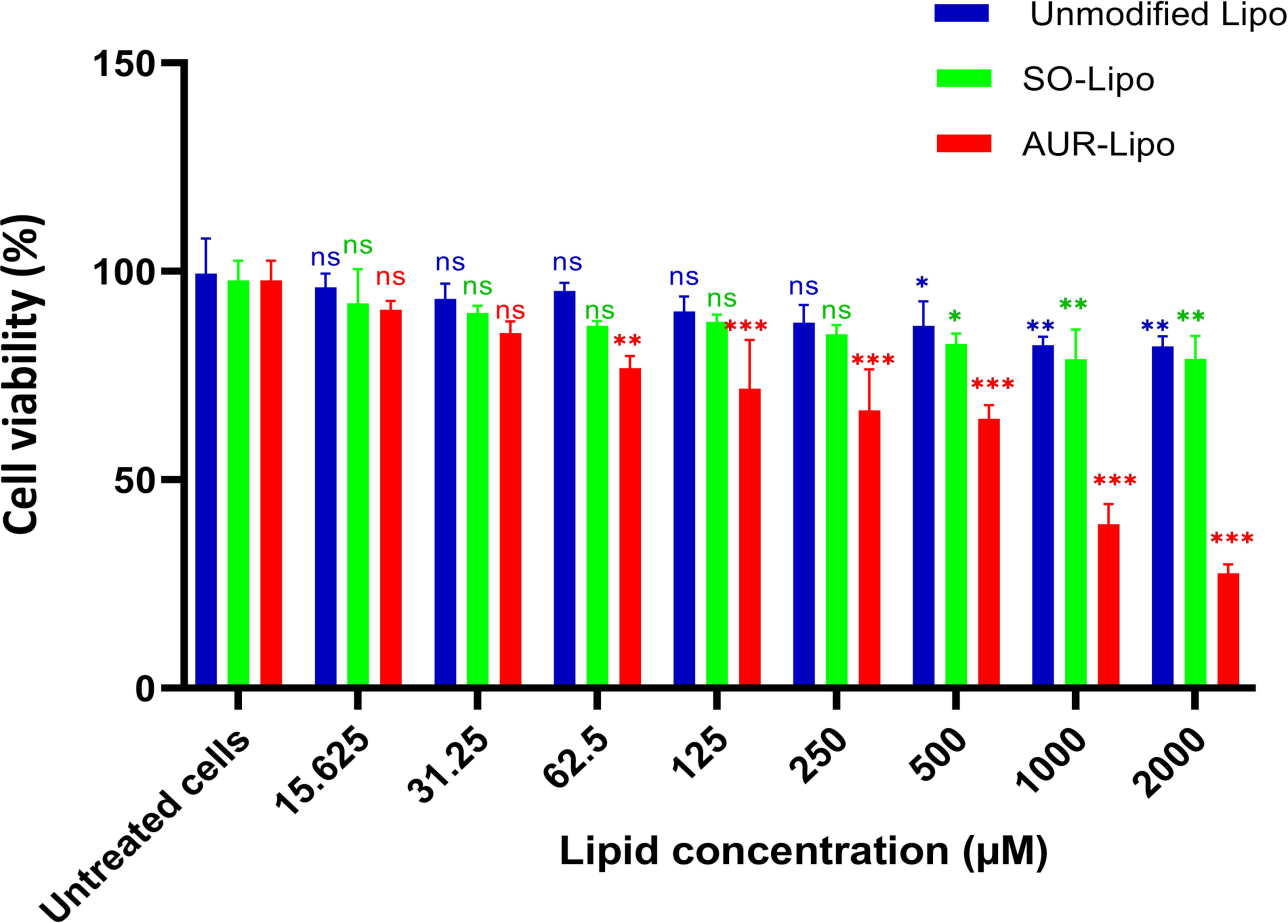
Cell viability of 4T1 cells following 48 h of treatment with varying concentrations (15.625 to 2000 µM) of Unmodified-Lipo, SO-Lipo, and AUR-Lipo. Data are presented as mean ± SD (*n* = 3). Statistical significance was assessed using one-way ANOVA followed by Tukey’s post-hoc test: *p < 0.05, **p < 0.01, ***p < 0.001, ns = not significant.

The cytotoxicity of Unmodified-Lipo, SO-Lipo, and AUR-Lipo was evaluated using a resazurin-based cell viability assay in a concentration range from 15.625 to 2000 µM, as illustrated in Figure 1. This assay is an established method for determining cell viability and based on the reduction of resazurin to resorufin by metabolically active cells. The data reveal that Unmodified-Lipo exhibited minimal cytotoxicity at lower lipid concentrations (15.625 to 250 µM), with no statistically significant differences compared to untreated control cells (*p* > 0.05). However, at a concentration of 500 µM, the cell viability slightly decreased, approaching significance (*p* = 0.051), indicating a potential threshold where lipid concentration may begin to impact cell viability. Significant cytotoxicity was observed at higher concentrations (1000 and 2000 µM) with *p*-values of 0.003 and <0.001, respectively, demonstrating a dose-dependent cytotoxic effect at elevated lipid concentrations. SO-Lipo displayed a similar trend, maintaining non-significant cytotoxicity from 15.625 to 250 µM (*p* > 0.05), supporting the biocompatibility of the SO modification at lower concentrations. A noticeable reduction in cell viability was observed at 500 µM (*p* = 0.017), indicating significant cytotoxicity. This trend was consistent at higher concentrations, with *p*-values of 0.002 at both 1000 and 2000 µM, showing that SO-Lipo remains relatively safe at lower concentrations but exhibits significant cytotoxicity at higher concentrations. In contrast, AUR-Lipo demonstrated significant cytotoxicity at much lower concentrations, with *p*-values of 0.009 and 0.001 at 62.5 µM and 125 µM, respectively. The cytotoxic effect became highly significant from 250 µM onwards (*p* < 0.001), indicating that AUR-Lipo is more cytotoxic at lower concentrations compared to Unmodified-Lipo and SO-Lipo. This aligns with previous studies that revealed the cytotoxic effect of AUR peptide on cancer cell lines [14–15]. Our results underscore the superior safety profile of SO-Lipo in comparison to AUR-Lipo, particularly at lower concentrations. The non-significant cytotoxicity observed when using SO-Lipo at concentrations up to 250 µM aligns with previous studies that have highlighted the safety and efficacy of SO in various formulations [16]. These findings highlight the cytocompatibility and safety potential of SO-Lipo as a promising candidate for drug delivery applications. The data support the use of SO as a safe modification in liposomal formulations, particularly in contexts where minimizing cytotoxicity is paramount.

### Cellular uptake

**Figure 2:**
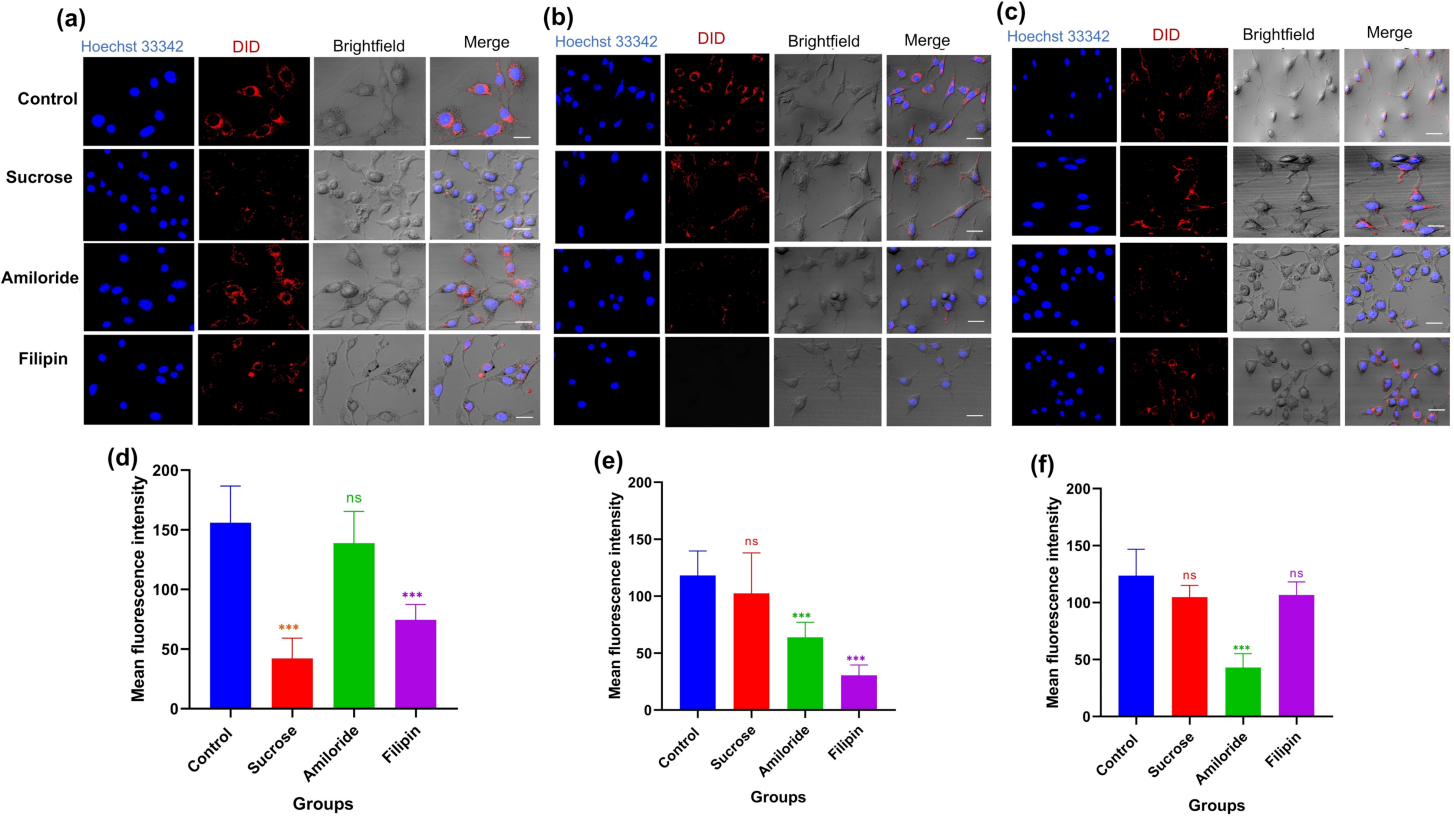
Cellular uptake of DiD-labeled liposomes in 4T1 cells with different endocytosis inhibitors. (a) Confocal images of Unmodified-Lipo in control cells and cells pretreated with sucrose, amiloride, or filipin inhibitors. Nuclei (blue), DiD-labeled liposomes (red), and merged bright-field images are shown. (b) Uptake of SO-Lipo under similar conditions. (c) Uptake of AUR-Lipo following the same inhibitor treatments. (d–f) Mean fluorescence intensity of DiD-labeled liposomes for (d) Unmodified-Lipo, (e) SO-Lipo, and (f) AUR-Lipo. Data are presented as mean ± SD. Statistical significance was determined using one-way ANOVA followed by Tukey’s post hoc test. ***p < 0.001, ns = not significant (*p* > 0.05).

The cellular uptake of DiD-labeled liposomes (Unmodified-Lipo, SO-Lipo, and AUR-Lipo) in 4T1 cells was investigated using different endocytic pathway inhibitors (Figure 2). For Unmodified-Lipo (Figure 2a,d), sucrose, which inhibits clathrin-mediated endocytosis, significantly reduced fluorescence intensity (*p* < 0.001), indicating that clathrin-mediated endocytosis is a primary pathway. Filipin, an inhibitor of caveolae-mediated endocytosis, also significantly reduced uptake (*p* < 0.001), suggesting the involvement of caveolae in the internalization of Unmodified-Lipo. Amiloride, which blocks macropinocytosis, had no significant effect (*p* < 0.99), indicating a negligible role for macropinocytosis in Unmodified-Lipo uptake. For SO-Lipo (Figure 2b,e), sucrose had no significant effect (*p* = 0.745), suggesting that SO shifts the uptake away from clathrin-mediated endocytosis. Amiloride significantly reduced uptake (*p* < 0.001), indicating that macropinocytosis is a key pathway for SO-Lipo, consistent with findings that fatty acids often use macropinocytosis for internalization [17]. Filipin significantly inhibited uptake (*p* < 0.001), showing that caveolae-mediated endocytosis also plays an important role in SO-Lipo uptake. These results align with reports that anionic particles undergo caveolae-mediated endocytosis, likely due to membrane modulation by SO [18–19]. For AUR-Lipo (Figure 2c,f), amiloride significantly reduced fluorescence (*p* < 0.001), confirming that macropinocytosis is the primary pathway for AUR-Lipo. This is consistent with previous studies of cationic antimicrobial peptides using micropinocytosis [19–20]. Filipin had no significant effect (*p* = 0.418), indicating a minor role for caveolae-mediated endocytosis, while sucrose also did not significantly affect uptake (*p* = 0.069), showing minimal involvement of clathrin-mediated endocytosis in AUR-Lipo uptake.

### Evaluation of endosomal escape

**Figure 3:**
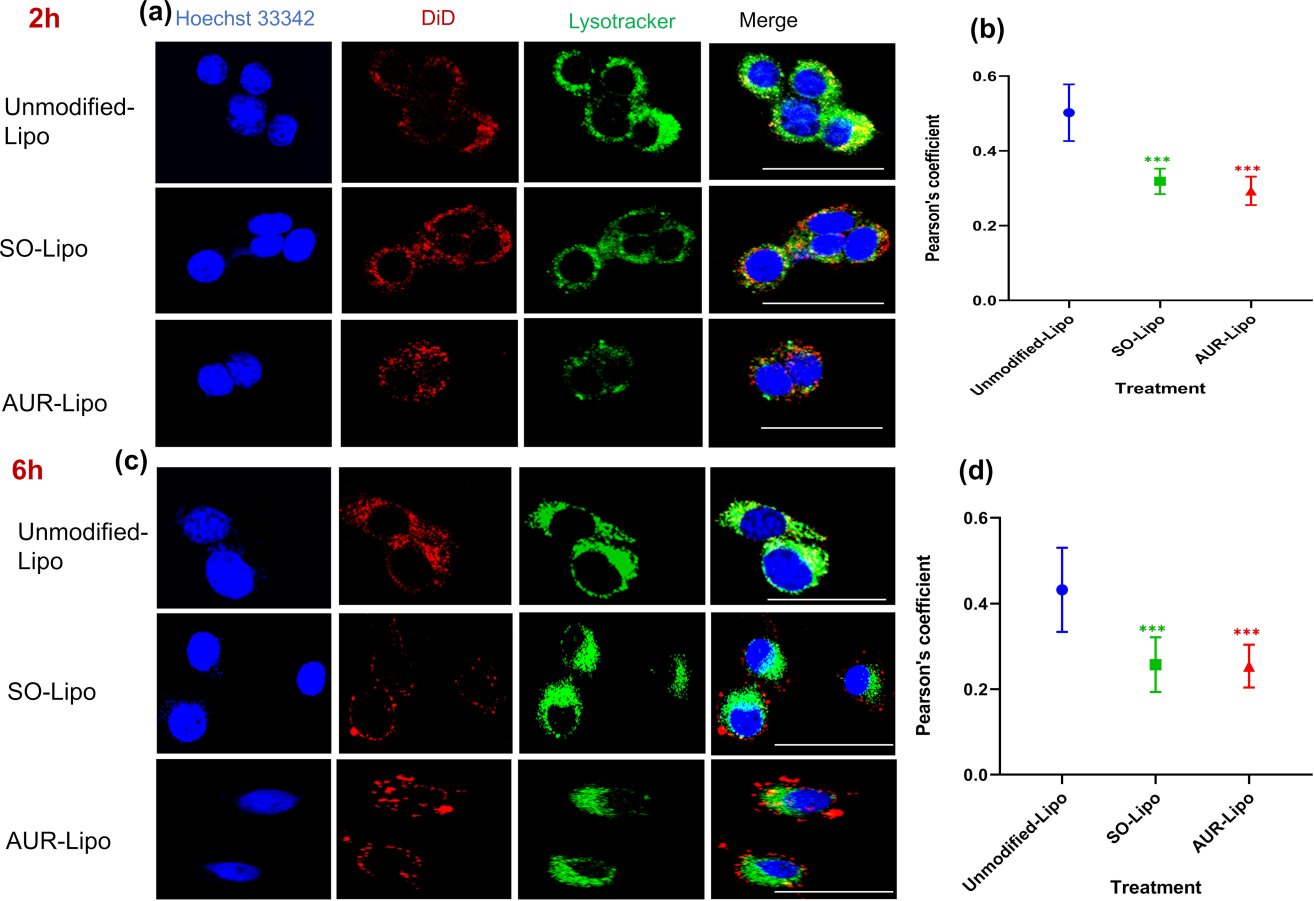
Colocalization analysis and endosomal escape efficiency of Unmodified-Lipo, SO-Lipo, and AUR-Lipo in 4T1 cells after 2 and 6 h. (a, c) Representative confocal images showing the colocalization of DiD-labeled liposomes (red) with LysoTracker-stained lysosomes (green) after 2 and 6 h of incubation. The scale bar represents 100 µm. (b, d) Pearson’s correlation coefficients after 2 and 6 h, quantifying the degree of colocalization. Data are expressed as mean ± standard deviation (SD) from three independent experiments. Statistical comparisons were conducted using one-way ANOVA with Tukey’s multiple comparison test, ***p < 0.001 versus unmodified liposomes.

Figure 3 illustrates the assessment of endosomal escape efficiency for Unmodified-Lipo, SO-Lipo, and AUR-Lipo after 2 and 6 h of incubation. The colocalization of the DiD-labeled liposomes (red) with LysoTracker-stained lysosomes (green) is a key indicator of whether the liposomes remain trapped within endosomal compartments or successfully escape into the cytosol. High colocalization appears as yellow in the merged images, signifying that the liposomes are primarily confined within the lysosomes.

To quantitatively assess the degree of colocalization, we employed Pearson’s correlation coefficient. A higher Pearson’s coefficient indicates a strong colocalization, suggesting that the liposomes are predominantly retained within lysosomal compartments. Conversely, a lower Pearson’s coefficient reflects weaker colocalization, indicating successful endosomal escape and greater distribution of the liposomes within the cytosol. At the two-hour mark (Figure 3a,b), Unmodified-Lipo displayed substantial yellow areas in the merged images, corresponding to a higher Pearson’s coefficient, indicating significant colocalization and, consequently, greater endosomal retention. In contrast, both SO-Lipo and AUR-Lipo showed reduced yellow regions and lower Pearson’s coefficients, suggesting more effective endosomal escape. Statistical analysis further substantiated these observations, revealing significant differences (*p* < 0.001) between Unmodified-Lipo and the modified liposomes (SO-Lipo and AUR-Lipo). At the six-hour time point (Figure 3c,d), Unmodified-Lipo continued to exhibit notable colocalization, as evidenced by the yellow areas, albeit slightly diminished. In contrast, SO-Lipo and AUR-Lipo demonstrated further decreases in Pearson’s coefficients, confirming their superior escape efficiency over time (*p* < 0.001). These results highlight the enhanced capacity of the modified liposomes to bypass endosomal entrapment, thereby improving intracellular delivery. Our findings underscore the significant impact of SO modification on liposomal formulations, particularly in enhancing endosomal escape. The efficient endosomal escape facilitated by SO is comparable to that achieved with AUR peptide, but with the added advantage of lower cytotoxicity. This combination of high efficacy and reduced toxicity positions SO as a highly attractive option for enhancing the delivery of a wide range of therapeutic agents.

### Lipid mixing and fusion

**Figure 4:**
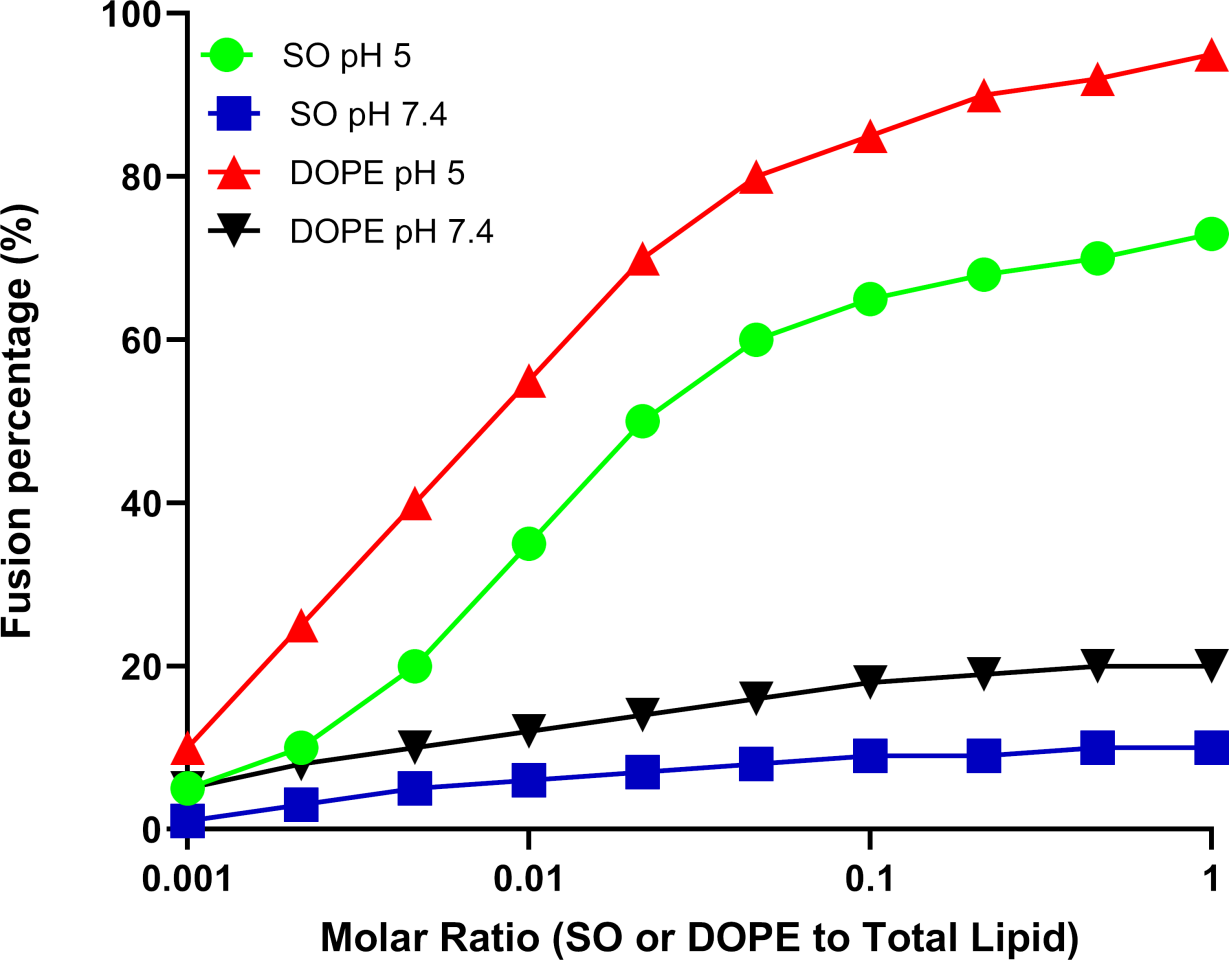
Fusion percentage of SO and DOPE at pH 5 and pH 7.4 measured via lipid mixing assay.

The fusogenic potential of SO and the well-known endosomal fusogenic agent dioleoylphosphatidylethanolamine (DOPE) was evaluated at varying concentrations expressed as the molar ratio of SO or DOPE to total phospholipid content, ranging from 0.001 to 1, under two pH conditions (pH 5 and pH 7.4). As shown in Figure 4, SO exhibited a concentration-dependent increase in membrane fusion at pH 5, reaching approximately 73% fusion at the highest tested molar ratio of 1. This suggests that SO facilitates lipid mixing and membrane fusion more effectively in an acidic environment, which is representative of endosomal conditions. In contrast, at pH 7.4, the fusion percentage for SO was significantly lower, peaking at only 10% at the highest concentration, highlighting the pH-sensitivity of SO’s fusogenic effect.

A similar trend was observed for the positive control, DOPE, which is known for its endosomal escape properties. DOPE exhibited enhanced fusion at pH 5, achieving over 95% fusion at molar ratio of 1. While DOPE’s fusion efficiency was highest under acidic conditions, it remained notably inactive at pH 7.4, showing minimal fusion (<20%) across all concentrations. These findings confirm that SO is effective at promoting membrane fusion under acidic conditions to achieve targeted fusogenicity, making it a promising candidate for enhancing endosomal escape in drug delivery applications.

### MD simulation of OLA and AUR interaction with lipid bilayers

**Figure 5:**
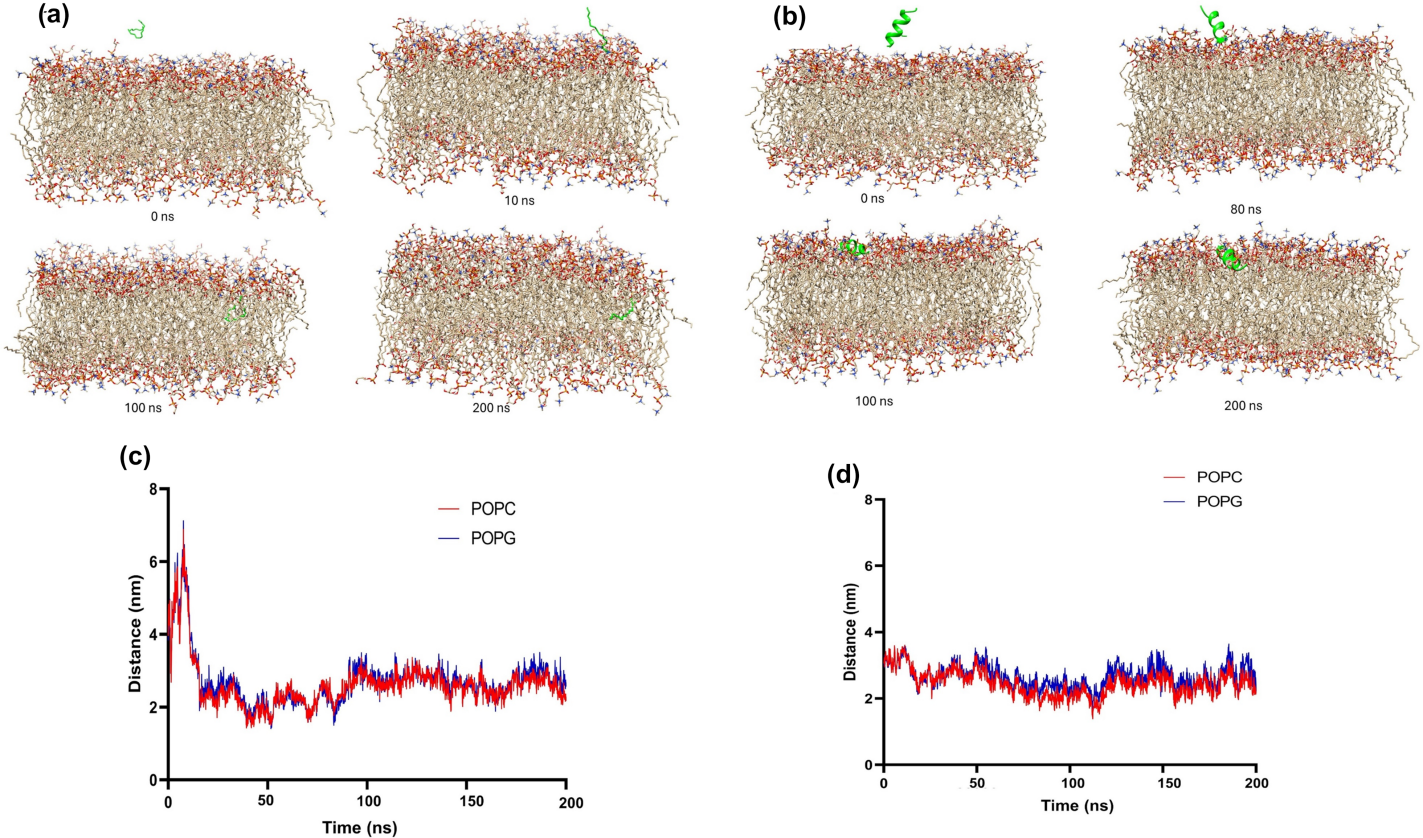
Molecular dynamics simulations of OLA and AUR interactions with a POPC/POPG lipid bilayer. (a) Snapshots showing the position of oleic acid (OLA) after 0, 10, 100, and 200 ns. (b) Snapshots depicting the position of aurein 1.2 peptide (AUR) after 0, 80, 100, and 200 ns. (c) Distance between the center of mass of POPC/POPG and OLA over 200 ns. (d) Distance between the center of mass of POPC/POPG and AUR over 200 ns.

The MD simulations depicted in Figure 5 provide a comprehensive analysis of the interaction dynamics between OLA and the AUR peptide with a model lipid bilayer composed of 1-palmitoyl-2-oleoyl-*sn*-glycero-3-phosphocholine (POPC) and 1-palmitoyl-2-oleoyl-*sn*-glycero-3-phospho-(1′-*rac*-glycerol) (POPG). These simulations, conducted over a 200 ns period, began with OLA and AUR positioned 6 and 4 Å above the membrane, respectively. As illustrated in Figure 5a, the time-resolved insertion of OLA occurs progressively. Initially (0 ns), OLA is situated above the membrane; however, after 10 ns, it has started to penetrate the bilayer. Notably, after 100 ns, OLA is significantly integrated within the membrane. At the end of the simulation (200 ns), OLA is thoroughly embedded, suggesting strong hydrophobic interactions that could potentially disrupt membrane integrity. This deep insertion is indicative of a substantial effect on membrane dynamics. In contrast, Figure 5b shows the interaction of AUR with the membrane. Unlike OLA, AUR remains above the membrane surface during the initial stages (0 ns), only beginning substantial interaction after around 80 ns. Subsequently, after 100 ns, AUR starts to insert into the membrane, and, after 200 ns, it is embedded within the bilayer, albeit less deeply than OLA. This gradual and less disruptive interaction is likely attributable to the amphipathic nature of AUR, which balances hydrophobic and hydrophilic interactions.

Further analysis, as shown in Supporting Information File 1, Figure S1, reveals that a crucial aspect of AUR’s interaction with the endosomal membrane is the necessity of protonation under acidic conditions. Specifically, at pH 5, protonation of the aspartic and glutamic acid residues in AUR’s structure is vital for effective membrane interaction. This protonation alters the peptide’s charge distribution, facilitating its association with the negatively charged lipid headgroups, thereby enhancing insertion and potential membrane perturbation. These findings are consistent with the work of Barrera et al., who demonstrated that protonation of aspartic and glutamic acid residues is crucial for the transmembrane insertion of peptides, particularly in acidic environments [21]. Our simulation data further indicate that, in the absence of protonation under these acidic conditions, there is no significant interaction between AUR and the membrane. This finding highlights the importance of the peptide’s protonation state regarding its membrane-binding affinity and subsequent insertion depth. Non-protonated AUR fails to engage effectively with the membrane, underscoring the role of electrostatic interactions in facilitating the peptide’s insertion into the lipid bilayer. The root-mean-square deviation (RMSD) analysis, shown in Supporting Information File 1, Figure S2, provides further insight into the structural stability of both OLA and AUR during their interaction with the membrane. The RMSD plots reveal that OLA exhibits more significant fluctuations initially, stabilizing at higher values, indicative of its dynamic integration into the bilayer. In contrast, AUR maintains lower RMSD values, suggesting a more controlled and stable interaction with the membrane. This difference in RMSD profiles further emphasizes the distinct interaction dynamics of OLA and AUR with the lipid bilayer, reinforcing the observed differences in their membrane integration and potential membrane modulation effects.

The distance plots in Figure 5c,d demonstrate the separation between the center of mass of the POPC and POPG components and the interacting molecules over time. For OLA, a sharp decrease in distance is observed within the first few nanoseconds, stabilizing at a lower value, indicating rapid insertion into the membrane. This interaction likely increases membrane fluidity and promotes the release of liposomal contents. Conversely, AUR shows a more gradual decrease in distance, reflecting its slower integration into the membrane. The final distance suggests that, while AUR interacts with the membrane, it does so in a less aggressive manner compared to OLA. The comprehensive simulation data underscore the distinct behaviors of OLA and AUR within the lipid bilayer. OLA’s rapid and deep integration reflects its potent ability to disrupt membrane structure, which likely leads to increased permeability. In contrast, AUR’s interaction is more controlled and surface-oriented and significantly influenced by its protonation state at acidic pH. The protonation-dependent insertion of AUR underscores a key mechanistic insight, namely, that effective membrane interaction at low pH values is contingent upon the peptide’s charge modulation, which is critical for endosomal escape processes. This interaction results in localized disruption at the membrane surface, specifically affecting the lipid headgroup region without deeply penetrating the membrane core. Thus, these findings emphasize the importance of molecular characteristics, such as hydrophobicity and charge state, regarding the interaction dynamics of OLA and AUR with lipid bilayers. OLA’s deeper integration contrasts with the more surface-level and protonation-dependent interaction of AUR, providing valuable insights into their potential applications in drug delivery and membrane modulation strategies.

### Analysis of membrane interactions with OLA and AUR

**Figure 6:**
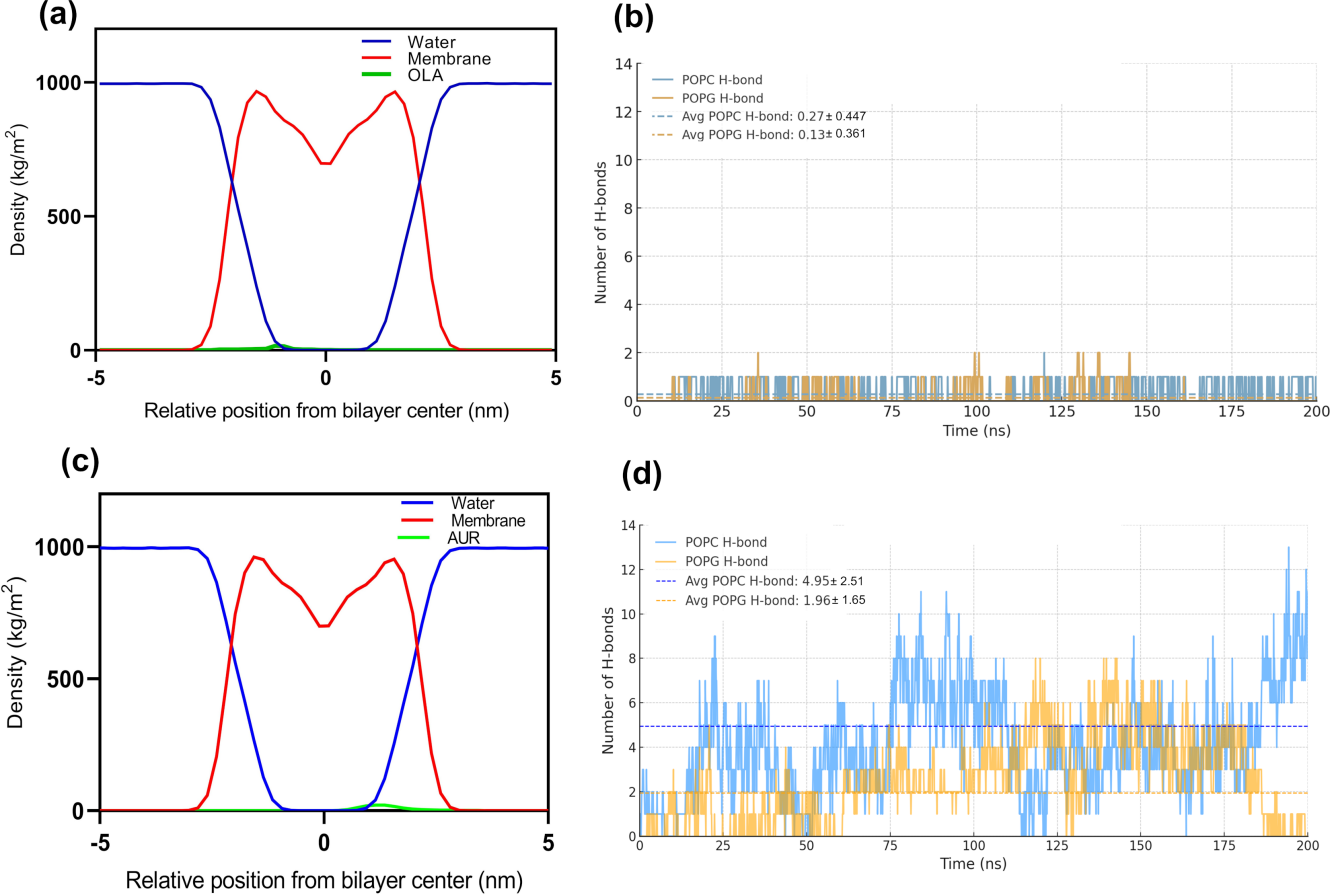
Density distribution and hydrogen bonding analysis over 200 ns. (a) Density distribution of water, membrane, and OLA. (b) Number of hydrogen bonds for POPC and POPG with OLA. (c) Density distribution of water, membrane, and AUR. (d) Number of hydrogen bonds for POPC and POPG with AUR.

This study investigates the behavior of OLA and AUR as they interact with a lipid bilayer model, providing insights into their potential roles in enhancing membrane permeability. MD simulations offer a detailed examination of these interactions, with Figure 6 highlighting critical aspects such as density distribution and hydrogen bonding over a 200 ns timescale.

The density profiles provide valuable insights into the spatial distribution and depth of insertion for both OLA and AUR within the lipid bilayer. In the case of OLA, the density profile (Figure 6a) reveals a pronounced peak near the bilayer center, suggesting deep integration into the hydrophobic core. This extensive penetration aligns with OLA’s hydrophobic nature, indicating strong interactions with the lipid tails. The overlap between OLA’s density and the membrane’s density profile suggests that OLA is well embedded within the membrane, potentially leading to significant perturbations in membrane structure and dynamics. In contrast, the density profile for AUR (Figure 6c) shows a peak near the water–membrane interface, indicating a more superficial insertion compared to OLA. AUR’s density decreases significantly toward the bilayer center, highlighting its preference for interacting with the membrane surface rather than penetrating deeply. This distribution reflects AUR’s amphipathic nature, where the peptide’s hydrophilic regions interact with the aqueous environment, while the hydrophobic regions associate with the lipid headgroups.

The hydrogen bond analysis (Figure 6b,d) complements these observations by quantifying the interactions between the molecules and the lipid components. For OLA, the analysis reveals a relatively low number of hydrogen bonds with the lipid headgroups, averaging 0.27 ± 0.447 with POPC and 0.13 ± 0.361 with POPG. This limited hydrogen bonding is expected given OLA’s hydrophobic nature, which favors interactions within the membrane’s core rather than with the polar headgroups. Conversely, AUR demonstrates a significantly higher propensity for hydrogen bonding, forming an average of 4.95 ± 2.51 bonds with POPC and 1.96 ± 1.65 bonds with POPG. This higher level of hydrogen bonding indicates strong interactions with the lipid headgroups, stabilizing AUR at the membrane surface. The temporal fluctuations in the number of hydrogen bonds suggest dynamic interactions, with periodic increases possibly reflecting reorientation events or transient binding within the membrane interface. AUR’s strong interaction with the membrane surface, coupled with its shallow insertion depth, suggests that it may play a role in modulating membrane surface properties without causing extensive disruption to the bilayer’s hydrophobic core. The differential interaction profiles and hydrogen bonding characteristics of OLA and AUR underscore their distinct mechanisms of action within lipid bilayers. OLA’s deep integration and limited hydrogen bonding suggest a capacity for significant membrane disruption, potentially enhancing drug delivery by increasing membrane fluidity and permeability. This observation is consistent with previous studies, such as those by Notman et al., which demonstrated through MD simulations that fatty acids like oleic acid can increase membrane fluidity and permeability in membranes composed of DPPC [22]. In contrast, AUR’s surface-level engagement and extensive hydrogen bonding highlight its role in stabilizing and potentially modulating the membrane interface, possibly facilitating controlled membrane perturbations such as localized thinning. These findings align with previous studies that have demonstrated AUR’s primary action through localized membrane destabilization [23]. Furthermore, our results correlate with studies proposing a detergent-like mechanism, where AUR induces high local curvature, disrupting membrane integrity without fully spanning the membrane [24].

**Figure 7:**
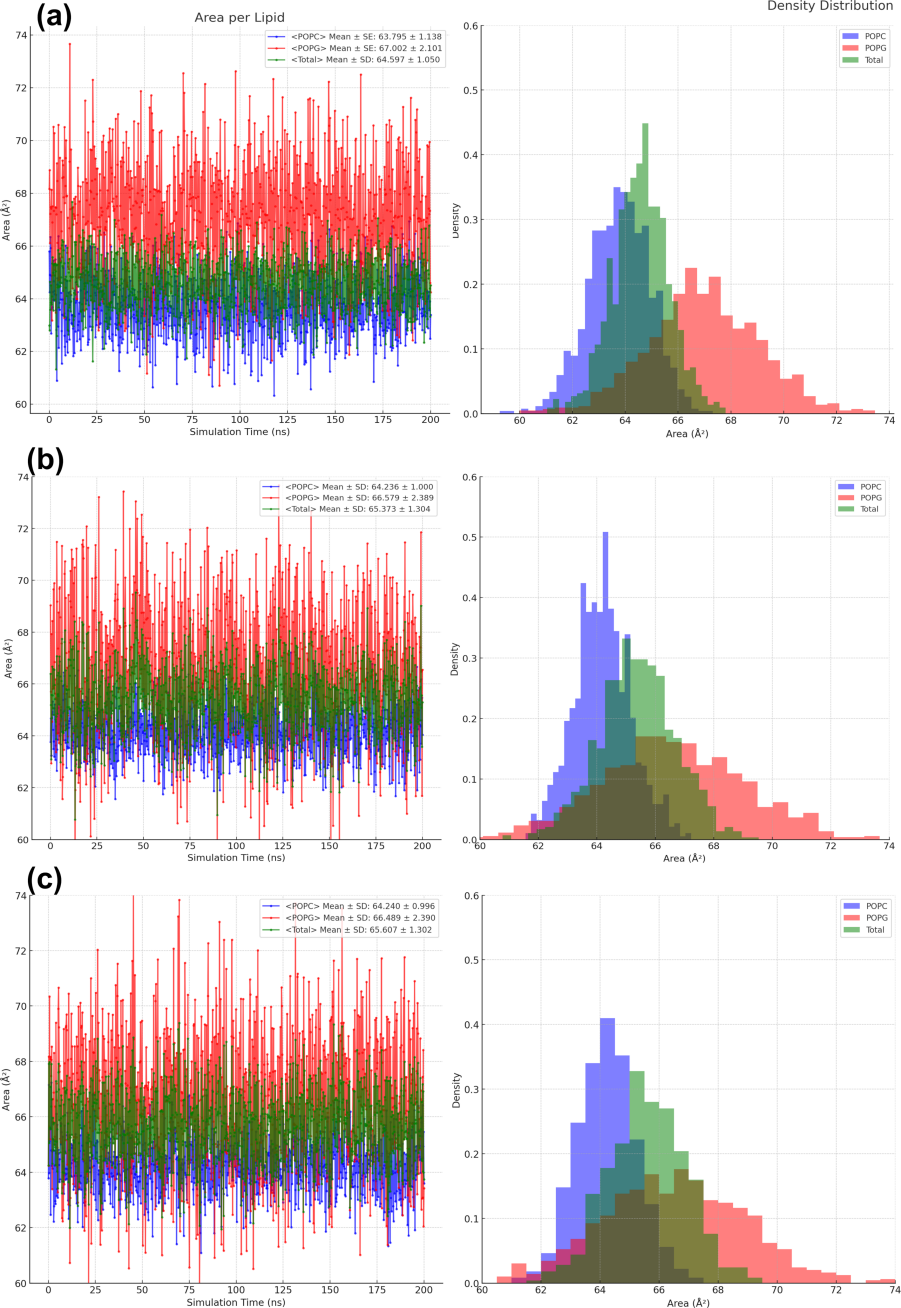
Area per lipid measurements over 200 ns. (a) Control. (b) OLA. (c) AUR. Each panel shows the area per lipid and the corresponding density distribution.

To further elucidate effects on membrane structure and dynamics, we assessed the area per lipid (APL) in Figure 7. It provides critical insights into the effects of OLA and AUR on the lipid packing density within the POPC/POPG bilayer over the 200 ns simulation period. In the control system, the APL values for POPC and POPG remain relatively stable, with mean values of approximately 63.795 ± 1.138 Å^2^ for POPC and 67.002 ± 2.101 Å^2^ for POPG. This consistency indicates a well-ordered bilayer with tightly packed lipids, which is in line with previous studies on similar lipid compositions, including those reporting typical APL ranges for POPC and POPG [25–26].

Upon the introduction of OLA and AUR, the APL values displayed notable fluctuations. For POPC, the mean APL increased slightly to 64.236 ± 1.000 Å^2^ (with OLA) and 64.240 ± 0.996 Å^2^ (with AUR). When considering the overall bilayer, the total mean APL increased, reaching 65.373 ± 1.304 Å^2^ (with OLA) and 65.607 ± 1.302 Å^2^ (with AUR), compared to the control value of 64.597 ± 1.050 Å^2^. As demonstrated by previous research, an increase in the area per lipid is generally indicative of looser packing of lipid molecules within the bilayer, correlating with increased membrane disorder [27]. These slight increases in APL suggest a minor loosening of the lipid packing, potentially reflecting a modest increase in membrane disorder. However, the similar mean APL values observed with both OLA and AUR agents suggest that the extent of this effect is comparable for both. This indicates that, while OLA and AUR may induce some degree of membrane disorder, the overall impact on lipid packing density is relatively small and does not significantly differ between the two agents.

**Figure 8:**
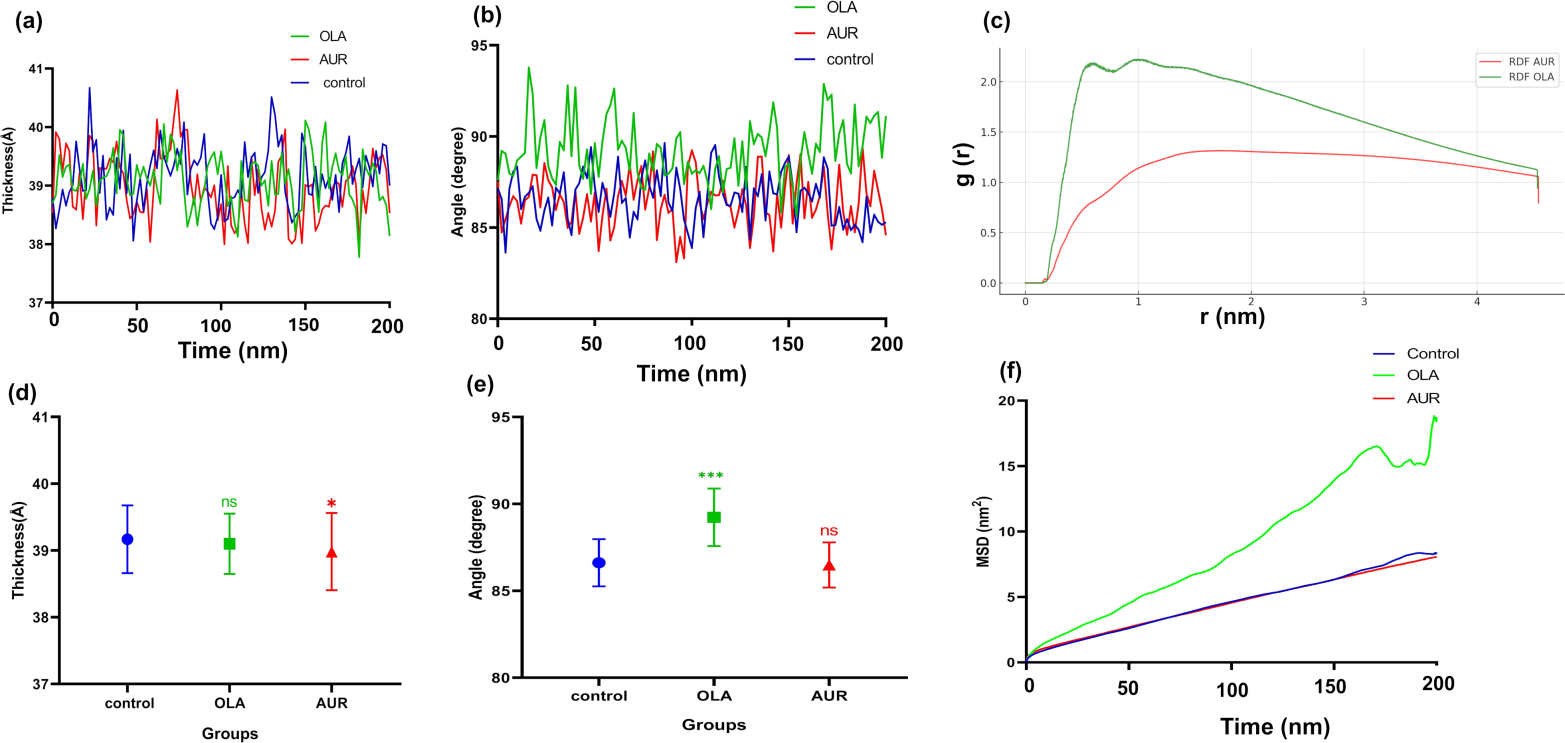
Membrane structural and dynamic properties over 200 ns. (a) Thickness of the membrane over time for control, OLA, and AUR. (b) Lipid tilt angle over time for control, OLA, and AUR. (c) Radial distribution function (RDF) for OLA and AUR. (d) Average membrane thickness for control, OLA, and AUR, analyzed using one-way ANOVA followed by Tukey’s post-hoc test; * indicates *p* < 0.05 compared to control. (e) Average lipid tilt angle for control, OLA, and AUR, analyzed using one-way ANOVA followed by Tukey’s post-hoc test; *** indicates *p* < 0.001 compared to control, ns indicates *p* > 0.05 (not significant). (f) Mean square displacement (MSD) for control, OLA, and AUR.

We further assessed the membrane’s structural and dynamic properties in the presence of OLA and AUR, focusing on membrane thickness, lipid tilt, radial distribution function (RDF), and mean square displacement (MSD), as shown in Figure 8.

The measurements of membrane thickness over time (Figure 8a) show distinct behaviors for OLA and AUR. Statistical analysis (Figure 8d) reveals a significant decrease in membrane thickness in the presence of AUR compared to the control (*p* = 0.021), indicating a notable structural impact. In contrast, OLA does not cause a significant change in membrane thickness compared to the control (*p* = 0.551). The reduction in thickness suggests that AUR’s interaction with the membrane may promote a more compact lipid arrangement, potentially due to increased packing density at the membrane surface. This effect could be attributed to AUR’s amphipathic nature, which facilitates specific binding interactions with lipid headgroups, thereby inducing closer lipid packing. Such interactions may involve hydrogen bonding with the lipid headgroups, especially with negatively charged lipids, as suggested by the higher number of hydrogen bonds formed by AUR compared to OLA. The lipid tilt angle data (Figure 8b) provides further insight into the structural impact of these molecules on the membrane. The statistical analysis of the average lipid tilt angle (Figure 8e) shows that OLA induces a significant increase in lipid tilt (*p* < 0.001), reflecting its disruptive influence on the membrane’s orderly structure. This increase in tilt suggests that OLA’s deep integration into the bilayer results in a more disordered and fluid membrane. In contrast, AUR does not cause a significant change in lipid tilt (*p* = 0.671), indicating that its interaction with the membrane does not disrupt the overall lipid arrangement. This suggests that AUR’s interactions are more localized to the membrane surface, maintaining the membrane’s structural order and integrity without inducing substantial changes in lipid orientation. The RDF data (Figure 8c) provides critical insights into the spatial distribution of OLA and AUR relative to the lipid components. OLA demonstrates a higher RDF peak, indicating a more structured and deeper association with the lipid molecules, which aligns with previous findings where OLA was shown to integrate deeply into the bilayer, promoting membrane fluidity and permeability. AUR shows a lower RDF peak, reflecting its surface-level interaction, consistent with its amphipathic nature that favors interactions with lipid headgroups rather than deep penetration into the hydrophobic core. The MSD analysis (Figure 8f) reveals that OLA significantly enhances the lateral mobility of lipids within the bilayer, indicative of a fluidizing effect. This increased mobility likely results from OLA’s ability to disrupt lipid packing through its deep insertion, facilitating greater lipid diffusion. In contrast, AUR does not significantly affect MSD, suggesting that its interaction primarily stabilizes the membrane without substantially altering the lateral dynamics, which is consistent with studies highlighting the role of amphipathic peptides in maintaining membrane integrity by interacting mainly with the headgroup region.

**Figure 9:**
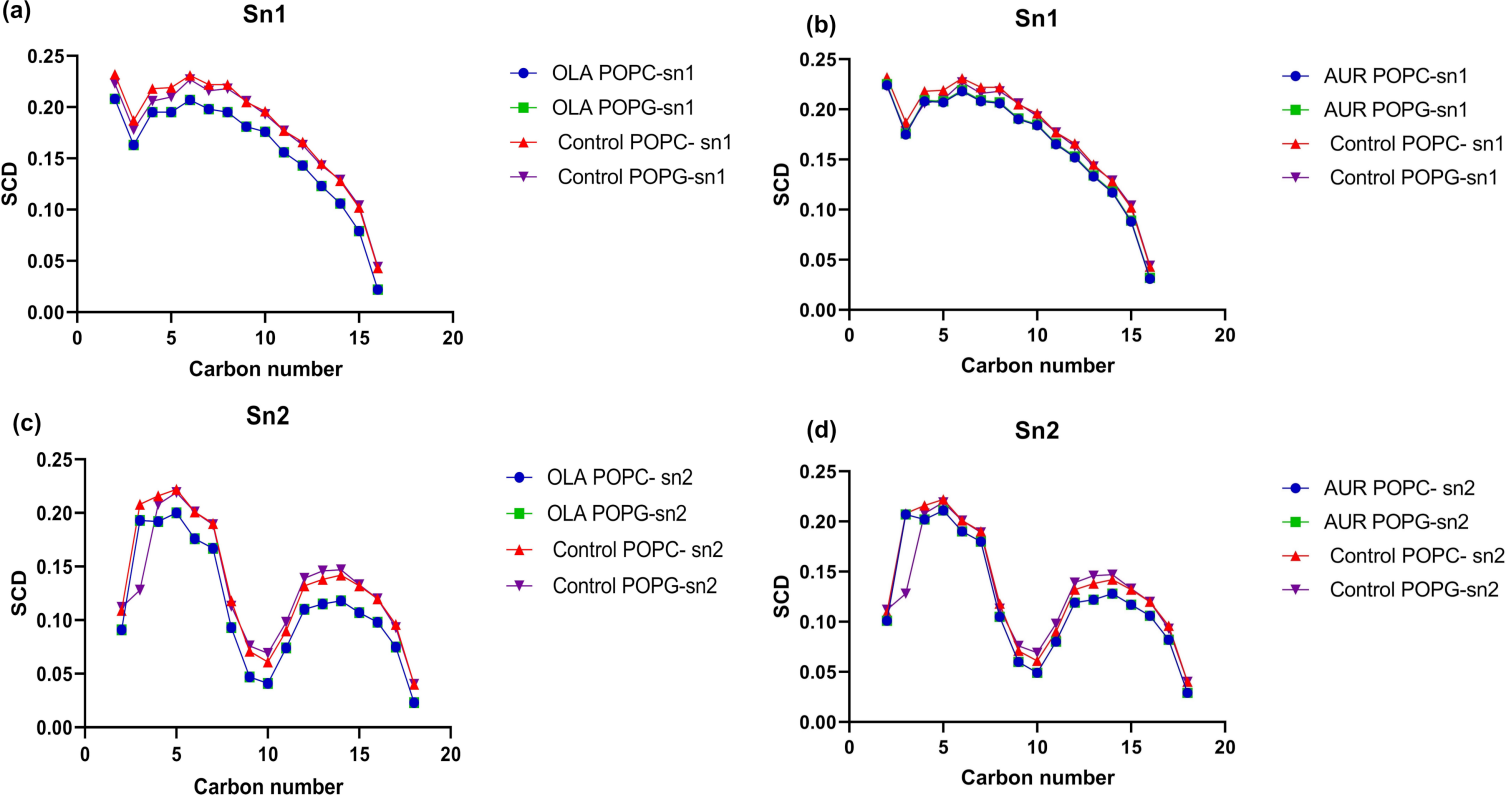
Deuterium order parameter (SCD) analysis for POPC and POPG lipid bilayers with OLA and AUR. (a) SCD for *sn*-1 position of POPC and POPG in control and OLA conditions. (b) SCD for *sn*-1 position of POPC and POPG in control and AUR conditions. (c) SCD for *sn*-2 position of POPC and POPG in control and OLA conditions. (d) SCD for *sn*-2 position of POPC and POPG in control and AUR conditions. Each panel shows the ordering of lipid acyl chains as indicated by the SCD for the specified carbon numbers.

In addition to the previous membrane analysis, the deuterium order parameter (SCD) in Figure 9 offers a detailed analysis of lipid alkyl chain alignment within the membrane, reflecting changes in membrane rigidity and fluidity. Higher SCD values correspond to a more structured, rigid membrane, while lower values indicate increased disorder and fluidity [28]. Our results indicate that OLA reduces SCD values across both *sn*-1 (Figure 9a,d) and *sn*-2 positions (Figure 9c,d) in POPC and POPG compared to the control, suggesting that OLA disrupts the packing of lipid molecules. This disruption leads to a more fluid membrane environment, as OLA’s deep integration within the bilayer diminishes lipid order throughout the membrane. AUR also causes a reduction in SCD values (Figure 9b,c), but to a lesser extent. The moderate decrease observed in AUR-treated membranes suggests that AUR induces localized disruptions at the membrane surface, affecting the lipid headgroup region without significantly altering the deeper hydrophobic core as evidenced by our previous membrane analysis.

Importantly, this study introduces an in silico model of endosomal escape with distinct mechanisms that could be harnessed for further design of novel endosomal escape agents. While AUR served as a positive control because of its well-known endosomal escape properties, our model revealed the critical role of protonation of aspartic and glutamic acid residues in endosomal environments. This protonation significantly enhances peptide–membrane interactions, suggesting a pathway for designing safer, more effective endosomal escape peptides. These insights not only expand our understanding of endosomal escape but also open avenues for the development of new strategies in targeted drug delivery.

### Proposed mechanism of sodium oleate-enhanced endosomal escape in drug delivery

In this study, we developed an innovative strategy to modify liposomes with SO to enhance their endosomal escape capabilities. By combining in vitro experiments with in silico MD simulations, we provide comprehensive insights into the interactions between SO and the endosomal membrane. Leveraging the pH-responsive properties of SO, our dual experimental approach deepens the understanding of the underlying mechanisms and paves the way for the design of next-generation endosomal escape agents. Upon endocytosis, SO-Lipo encounter the acidic environment of the endosome, triggering the protonation of SO into OLA. This protonation significantly alters the physicochemical properties of the liposomes, notably reducing their negative zeta potential, as previously described in Table 1. The reduction in surface charge diminishes electrostatic repulsion between the liposomes and the endosomal membrane, thereby enhancing hydrophobic interactions. These changes are critical for facilitating the structural perturbations required for efficient endosomal escape.

In vitro colocalization studies demonstrated a significant reduction in the colocalization of SO-Lipo with lysosomal markers compared to unmodified liposomes, as quantified by the Pearson’s correlation coefficients. The lower Pearson’s coefficients indicate that SO-Lipo are less confined within endo-lysosomal compartments, suggesting enhanced escape into the cytosol. This empirical evidence correlates with our MD simulation findings, providing a molecular basis for the observed decrease in colocalization. Protonated OLA was shown in simulations to integrate deeply into the lipid bilayer of the endosomal membrane, resulting in increased lipid disorder. This was evidenced by changes in the SCD, reflecting disruption in the orderly packing of lipid acyl chains. The incorporation of OLA disrupts this packing, contributing to increased membrane fluidity. Simultaneously, the simulations revealed enhanced lateral mobility of lipids, as indicated by increased MSD values. Collectively, these changes destabilize the bilayer structure, facilitating endosomal membrane permeation and priming the bilayer for the structural rearrangements required for membrane fusion. The increased membrane fluidity and lipid disorder observed in the simulations provide a mechanistic explanation for the decreased colocalization observed in vitro. As the membrane becomes more disordered and fluid due to OLA integration, it becomes more permeable, allowing the liposomes to fuse with the endosomal membrane and release their contents into the cytosol. This fusion and escape reduce the likelihood of the liposomes remaining within endo-lysosomal compartments, which is reflected by the lower Pearson’s correlation coefficients.

**Figure 10:**
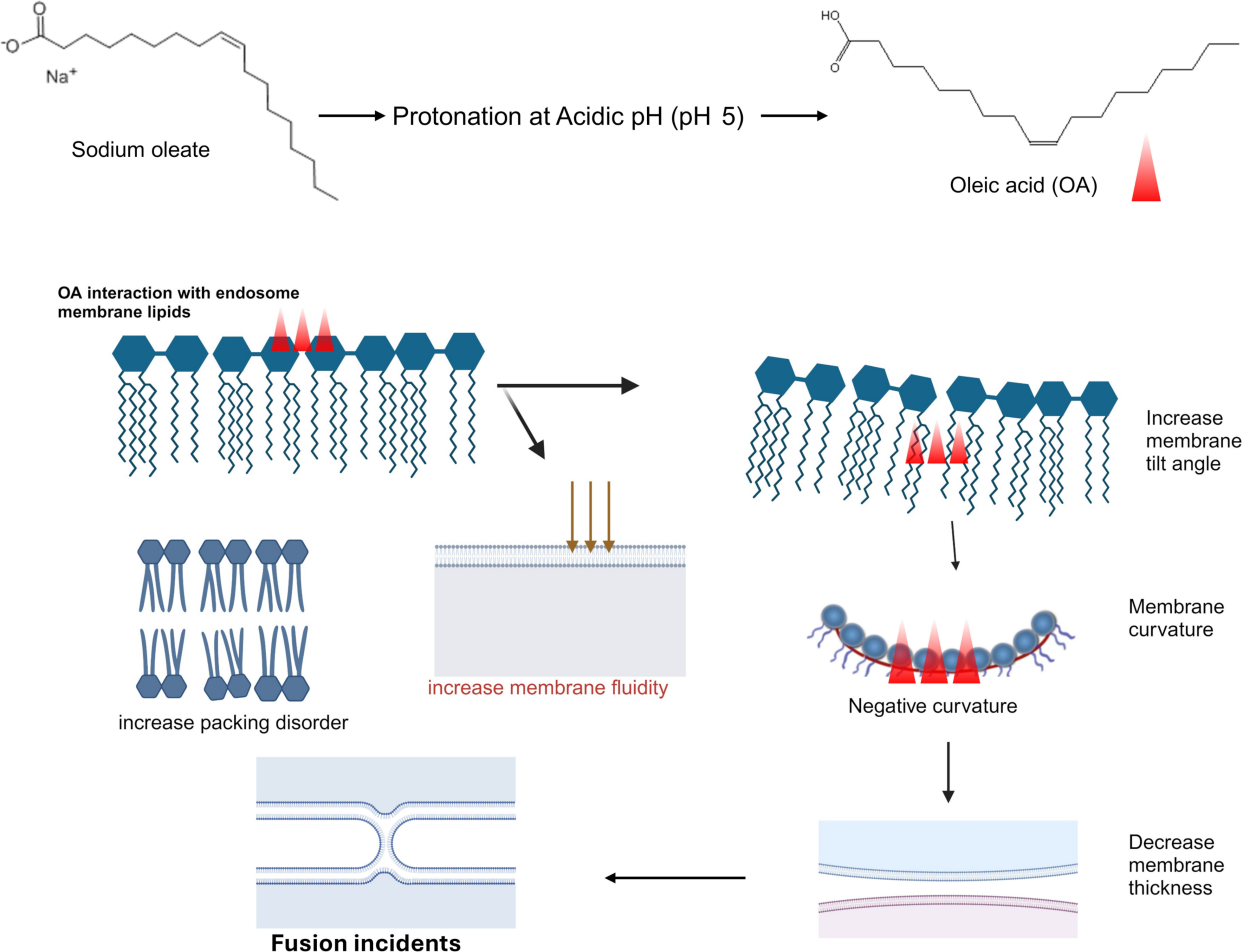
Mechanistic illustration of sodium oleate-mediated endosomal escape. Created in BioRender. Mudhakir, D. (2024) https://BioRender.com/x99g891. This content is not subject to CC BY 4.0.

Interestingly, in vitro lipid mixing assays further corroborated these findings by demonstrating that SO exhibits significant pH-dependent fusogenicity. Specifically, over 70% membrane fusion was achieved under acidic conditions, compared to only 10% at neutral pH. This result underscores the critical role of SO protonation in enhancing fusogenic properties. The lipid mixing assays provide empirical evidence of membrane fusion events facilitated by SO-Lipo under acidic conditions. To understand the molecular basis of this enhanced fusion, we analyzed both short-term and extended MD simulations focusing on lipid tilt, membrane thinning, and curvature generation. The short-term MD simulations, conducted over 200 ns, provided initial insights. Although they showed a decrease in membrane thickness upon the incorporation of OLA into the bilayer, this change was not statistically significant. However, a significant increase in lipid tilt was observed, indicating that OLA has a pronounced effect on membrane structure even in the short term. Extended simulations, incorporating 20% OLA over 1 μs (Supporting Information File 1, Figure S3), revealed significant structural changes in the membrane. The control membrane maintained a thickness of 39.17 ± 0.51 Å, while the OLA-incorporated membrane exhibited a reduced thickness of 35.91 ± 0.42 Å. This pronounced thinning effect was accompanied by further increases in lipid tilt angles. Lipid tilt plays a crucial role in membrane fusion events. As bilayers approach each other during the fusion process, the area per lipid molecule increases, causing individual lipid molecules to tilt. This tilt facilitates the splaying of lipid tails, allowing them to insert into the opposing bilayer and initiate fusion [29]. The increased lipid tilt observed in our simulations leads to the generation of negative curvature in the membrane, a critical factor for the formation of fusion intermediates such as stalks. Membrane thinning observed in the extended simulations reduces the physical distance between opposing bilayers, lowering the energy barrier for membrane fusion by bringing the bilayers closer together. Thinner membranes are more susceptible to curvature and deformation, which are essential for fusion events. The combination of increased lipid tilt and membrane thinning facilitates the necessary curvature generation for fusion, aligning with the high levels of fusion observed in the in vitro lipid mixing assays under acidic conditions. These curvature changes align with previous studies suggesting that the kinked structure of OLA, resulting from its cis double bond, increases the propensity to induce negative curvature within the bilayer of DOPC [10]. The fusion process is intrinsically linked to the shape and behavior of lipids within the bilayer. Lipids with non-lamellar and non-bilayer-forming tendencies, such as those exhibiting conical or inverted conical shapes, play critical roles in facilitating fusion by promoting the membrane curvature required for the process [27,30]. As illustrated in Figure 10, the proposed mechanism by which SO-Lipo facilitates endosomal escape involves the protonation of SO in the acidic endosomal environment, initiating a cascade of biophysical changes consistently observed in both in vitro experiments and MD simulations. Protonation reduces the negative surface charge of the liposomes, decreasing electrostatic repulsion and enhancing hydrophobic interactions with the endosomal membrane. The integration of protonated OLA increases membrane fluidity and disrupts lipid packing, leading to increased lipid tilt and membrane thinning, which ultimately cause endosomal membrane fusion.

This mechanism is evidenced by in vitro experiments and further elucidated by MD simulations, which demonstrate increased lipid tilt, membrane thinning, and the induction of membrane curvature. These findings are consistent with previous studies describing the fusion process as involving interconnected events, including increased lipid tilt, membrane thinning, and the development of negative curvature [31–33]. These findings establish SO as a promising endosomal escape enhancer, offering significant potential for improving the efficiency of liposomal drug delivery systems. By combining in vitro experimentation with in silico MD simulations, this study underscores the importance of integrated approaches in unraveling the complex mechanisms of intracellular trafficking. Further in vivo validation is necessary to confirm the efficacy of SO in various therapeutic nanotherapies, potentially broadening its application in nanomedicine. This work provides a robust framework for the rational design of liposomal formulations with optimized endosomal escape capabilities, advancing the development of next-generation drug delivery systems.

## Conclusion

This study provides a comprehensive analysis of the mechanisms through which SO-Lipo facilitate endosomal escape. Our findings demonstrate that SO-Lipo significantly enhance cytosolic delivery in 4T1 triple-negative breast cancer cells, as evidenced by a marked reduction in colocalization with lysosomal markers. The enhanced endosomal escape capability of SO-Lipo is primarily driven by its fusogenic interactions with the endosomal membrane, effectively overcoming the barrier of endosomal entrapment. MD simulations further elucidated the protonation-dependent mechanism of SO, which converts into OLA in the acidic endosomal environment. This conversion enables deep integration into the endosomal membrane, increasing membrane fluidity, altering lipid tilt, and facilitating fusion events. In contrast, our in silico endosomal escape model revealed a distinct mechanism for the AUR peptide. It induces localized membrane disruptions in a detergent-like manner, driven by the protonation of key residues (aspartic and glutamic acid), which is crucial for its interaction with the endosomal membrane. These findings highlight the importance of protonation in modulating endosomal escape pathways and underscore the unique mechanisms employed by SO and AUR. The distinct advantage of SO lies in its ability to achieve effective endosomal escape while maintaining a safer cytotoxicity profile compared to AUR. This study introduces SO-Lipo as a promising approach for developing liposomal carriers with enhanced endosomal escape capabilities. Future in vivo studies are necessary to validate the therapeutic potential of SO-Lipo, potentially advancing the field of targeted drug delivery and nanomedicine.

## Experimental

### Materials

The lipids 1,2-dimyristoyl-*sn*-glycero-3-phosphocholine (DMPC, CAS No. 18194-24-6), 16:0 Liss Rhod PE (rhodamine B-PE, CAS No. 384833-01-6), NBD-PE (CAS No. 384832-99-9), and 1,2-dioleoyl-*sn*-glycero-3-phosphoethanolamine (DOPE, CAS No. 4004-05-1) were purchased from Avanti Polar Lipids (Alabaster, AL, USA). Cholesterol (CAS No. 57-88-5, ≥99% purity), 4-(2-hydroxyethyl)-1-piperazineethanesulfonic acid (HEPES, CAS No. 7365-45-9, ≥99.5% purity), resazurin (CAS No. 62758-13-8, ≥98% purity), and sodium oleate (CAS No. 143-19-1, ≥97% purity) were obtained from Sigma-Aldrich (St. Louis, MO, USA). Phosphate-buffered saline (PBS, Catalog No. 10010-023), Dulbecco’s modified Eagle’s medium (DMEM, high glucose; Catalog No. 11965092), fetal bovine serum (FBS, Premium Grade; Catalog No. A5670701), penicillin-streptomycin (Catalog No. 15140122), trypan blue (Catalog No. 15250061), and trypsin-EDTA solution (Catalog No. 25200056) were supplied by Gibco, Thermo Fisher Scientific (Waltham, MA, USA). Fluorescent dyes LysoSensor Green DND-189 (Catalog No. L7535) and Hoechst 33342 (Catalog No. H3570) were also procured from Thermo Fisher Scientific. The stearylated aurein 1.2 peptide (≥95% purity) was synthesized by GL Biochem (Shanghai, China). 4T1 mouse mammary carcinoma cells (ATCC^®^ CRL-2539) were generously provided by Dr. Muhammad Hasan Bashari, Faculty of Medicine, Padjadjaran University, Indonesia.

### Preparation of liposomal formulations

In this study, we engineered three distinct liposomal formulations to investigate mechanisms of endosomal escape, namely, unmodified-Lipo, SO-Lipo, and AUR-Lipo. The base composition for the unmodified liposomes consisted of DMPC and cholesterol, mixed in a molar ratio of 9:1, yielding a total lipid concentration of 2.7 mM. The SO-Lipo variant was enriched with 10 mol % sodium oleate incorporated into the lipid blend before the liposome assembly process. Divergently, the fabrication of AUR-Lipo involved the post-integration of 135 µM stearylated aurein 1.2 peptide into pre-assembled liposomes, using HEPES buffer as the medium. This approach ensures the anchorage of the peptide to the liposome membrane surface. Synthesis of the liposomal formulations was conducted via a refined thin-film hydration technique, as inspired by existing protocols [34]. Initially, the lipids were dissolved in a chloroform and methanol solution (2:1 v/v), which was then systematically evaporated under nitrogen to form a thin lipid film. This film was rehydrated with HEPES buffer at pH 7.4, yielding multilamellar liposomes. Subsequent sonication in a bath sonicator for 5 min at room temperature facilitated the conversion to unilamellar vesicles. To standardize the size distribution, the sonicated dispersion underwent five cycles of extrusion through a 100 nm polycarbonate membrane using an Avanti Mini-Extruder. The finalized liposomal formations underwent a comprehensive characterization, assessing size, PDI, and zeta potential through photon correlation spectroscopy, employing a Delsa Nano C Particle Size Analyzer (Beckman Coulter, West Sacramento, CA, USA). The analysis was conducted in two different pH environments, that is, at pH 7.4 and at pH 5.0; the latter was achieved by adjusting the solution with 0.1 M HCl.

### Resazurin-based cell viability assay

To evaluate the cytotoxicity of the liposomal formulations, we employe the resazurin-based cell viability assay, a method widely employed and deemed reliable for assessing cell metabolic activity, which serves as an indicator of cell viability. The process involves the transformation of resazurin (initially blue and non-fluorescent) into resorufin (pink and fluorescent) by metabolically active cells, facilitating the measurement of cell viability [35–36]. Briefly, 4T1 cells were cultured in DMEM supplemented with 10% FBS and 1% penicillin–streptomycin in a humidified incubator at 37 °C with 5% CO_2_ until they reached an optimal confluence. These cells were then trypsinized and placed in 96-well plates at a concentration of 15,000 cells per well in 100 µL of complete medium. Subsequently, the cells were allowed to adhere and proliferate for 24 h before undergoing any treatments. Following the incubation period, the culture medium was aspirated, and the cells were exposed to 100 µL of liposomal formulations, including SO-Lipo, AUR-Lipo, and unmodified Lipo, at various concentrations ranging from 15.625 to 2000 µM, diluted in complete medium. Cells that did not receive any treatment were utilized as the negative control. The cells were then subjected to the treatments for 48 h under standard culture conditions. Following the treatment period, the medium was aspirated, and a resazurin solution (0.1 mg/mL) prepared in complete medium was added to each well (100 µL per well). The plates were incubated for an additional 3 h at 37 °C to facilitate the reduction of resazurin by the cells. Absorbance was subsequently measured at 570 and 600 nm using a microplate reader, providing data on cell viability.

### Cellular uptake assay

4T1 triple-negative breast cancer cells were seeded at a density of 2 × 10^5^ cells/well in 35 mm confocal dishes and incubated for 24 h at 37 °C with 5% CO_2_. To block specific endocytic pathways, the cells were pretreated with pathway inhibitors for 30 min at 37 °C. These inhibitors were sucrose (450 mM) to inhibit clathrin-mediated endocytosis, amiloride (50 µM) to block macropinocytosis, and filipin (5 µg/mL) to inhibit caveolae-mediated endocytosis. After inhibitor treatment, cells were incubated with DiD-labeled liposomes for 2 h at 37 °C. Post-treatment, cells were washed with cold PBS and stained with Hoechst 33342 (1 μg/mL) to visualize the nuclei. Confocal microscopy (Olympus FV-1200 Tokyo, Japan) was used to capture images of cellular uptake, and the mean fluorescence intensity was quantified using Fiji-ImageJ software for semi-quantitative analysis.

### Evaluation of endosomal escape efficiency through subcellular colocalization analysis

In order to assess the intracellular transport of various nanoformulations, subcellular colocalization was carried out following the methodology proposed by Torres-Vanegas et al. with adaptations [37]. Specifically, Unmodified-Lipo, SO-Lipo, and AUR-Lipo were utilized, incorporating the fluorescent membrane probe Vybrant DiD into the lipid bilayers during the preparation of liposomes. Cultured 4T1 breast cancer cells were seeded at a density of 1 × 10^5^ cells/well in glass-bottom 35 mm confocal dishes and incubated for 24 h at 37 °C. Subsequently, the cells were treated with the DiD-labeled liposomes for 2 and 6 h. After the incubation period, LysoTracker Green DND-189 (100 nM) was administered for 30 min to visualize lysosomes through fluorescence, while Hoechst 33342 (1 μg/mL) was applied for 10 min to visualize the nuclei. Following triple rinsing with cold PBS, the cells were visualized using confocal laser scanning microscopy (Olympus FV-1200 Tokyo, Japan). Pearson’s correlation coefficient was computed using Fiji-ImageJ software to assess the degree of colocalization between DiD-liposomes and LysoTracker, providing a semi-quantitative indicator of endosomal escape dynamics over time.

### Lipid mixing assay

Liposomes were prepared using the thin-film hydration method to simulate endosomal vesicles [38]. A mixture of POPC and POPG lipids in a 3:1 molar ratio was dissolved in chloroform/methanol (2:1 v/v), followed by solvent evaporation under a nitrogen stream to form a thin lipid film. The dried lipid film was subsequently hydrated with either acetate buffer at pH 5.0 (to mimic the acidic environment within endosomes) or PBS at pH 7.4 (to simulate physiological conditions), resulting in a final lipid concentration of 100 µM. Large unilamellar vesicles (LUVs) with an approximate diameter of 200 nm were produced by vortexing and sonication. Fluorescent probes, NBD-PE (donor) and rhodamine B-PE (acceptor), were incorporated into the lipid bilayer at a concentration of 0.3 mol % relative to the total lipid content. These liposomes were designed to closely mimic endosomal membranes, providing an ideal model for assessing fusogenic interactions.

The fusogenic potential of SO was evaluated using a modified version of the protocol described by Lonez et al., with specific adjustments to suit the experimental design [39]. Briefly, liposome samples were incubated with varying concentrations of SO defined as the molar ratio of SO to total phospholipid content, ranging from 0.001 to 1. The same concentrations were used for the positive control, DOPE, to enable direct comparison of their fusogenic effects. Incubations were carried out at 37 °C for 60 min to facilitate membrane fusion. Fluorescence resonance energy transfer (FRET) was used to monitor these fusion events, employing NBD-PE and rhodamine B-PE as the FRET donor–acceptor pair. Fluorescence measurements were recorded using a Synergy H1 multimode reader (BioTek Instruments, Winooski, VT, USA), with excitation set at 470 nm for NBD and emission spectra collected from 500 to 600 nm. Specifically, fluorescence intensities at 530 nm (NBD emission) and 580 nm (rhodamine B emission) were analyzed to monitor changes in FRET efficiency, indicative of lipid mixing and membrane fusion. As lipid mixing occurred, dilution of the membrane-incorporated fluorophores resulted in a decrease in the FRET signal from rhodamine B and an increase in NBD fluorescence, signaling membrane fusion. Control experiments were conducted using liposomes prepared without the tested agent (SO) or the positive control (DOPE) to establish baseline FRET efficiency. These controls provided a reference for fluorescence measurements in the absence of any fusogenic agents. To determine maximum fluorescence, 0.1% (v/v) Triton X-100 was added to fully permeabilize the liposomes, resulting in the complete release of encapsulated fluorophores. The fluorescence intensity after Triton X-100 treatment was recorded as the maximum fluorescence (*F*_Triton_).

The percentage of membrane fusion was calculated based on NBD fluorescence at 530 nm (FNBD) using the following formula:


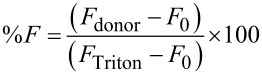


where *F*_donor_ represents the fluorescence intensity of NBD during the experiment, *F*_0_ is the baseline fluorescence from the control sample without the tested fusogenic agent, and *F*_Triton_ is the maximum fluorescence obtained after Triton X-100 treatment.

### In silico structure preparation

The OLA molecular structure was sourced from the crystal structure available in the Research Collaboratory for Structural Bioinformatics Protein Data Bank (RCSB PDB) with the ligand identifier OLA. Following retrieval, the structure was subjected to geometry optimization and energy minimization using the PM6 semi-empirical method in Gaussian 09 software. For the computational modeling of the Aurein 1.2 peptide, we employed the ab initio modeling capabilities of the Iterative Threading ASSEmbly Refinement (I-TASSER) suite, which facilitated the determination of the peptide’s primary sequence and the prediction of its tertiary structure through threaded alignment and iterative structural refinement [40].

### MD simulation

We modeled the endosomal membrane by constructing a lipid bilayer using the CHARMM-GUI interface [41]. The control system, designed to represent the endosomal membrane, comprised a biphasic lipid bilayer with a 3:1 molar ratio of POPC to POPG, totaling 256 lipid molecules. Each leaflet contained 128 lipids, creating an asymmetrical distribution that mirrors the natural structure of endosomal membranes, as documented in previous research [42]. The OLA molecule was initially positioned approximately 6 Å above the membrane model to simulate the onset of its interaction with the lipid bilayer. Similarly, the AUR peptide, in its PDB-acquired structural conformation, was placed 4 Å above the bilayer. We investigated the peptide under two protonation conditions, that is, one representing a neutral pH, where aspartic acid at position 4 and glutamic acid at position 11 remained unprotonated, and another mimicking the acidic environment of the endosome (pH 5), where these residues were protonated. After assembling the structure, the system was solvated using the TIP3P water model, and ionic balance was maintained by adding Na^+^ and Cl^−^ ions as necessary. System stability post-solvation was ensured through energy minimization, employing the Steepest Descent integrator until the force on all atoms fell below the energy minimization threshold of 100.0 kJ/mol·nm. To prepare the system thoroughly for the main simulation, we conducted six equilibration stages of increasing rigor, culminating in the final two stages, each lasting 2 ns, to allow the system to reach a stable equilibrium. The molecular dynamics simulations were then run for 200 ns using the GROMACS 2021.5 software suite. The leapfrog algorithm was utilized for integration with a time step of 2 fs, and the simulations were carried out under the NPT (isothermal–isobaric) ensemble. The CHARMM36m force field was selected for its precision in representing interatomic interactions. To replicate physiological conditions, the temperature was maintained at 310 K using the Nosé–Hoover thermostat [43]. The isotropic pressure was consistently held at 1 atm with the Parrinello–Rahman barostat, featuring a time constant of 1.0 ps and a compressibility factor of 4.5 × 10^−5^ bar^−1^ [44].

### Evaluation of membrane interactions

To investigate the complex interactions between OLA, AUR peptide, and the lipid bilayer, we conducted a comprehensive analysis using various advanced methodologies. The initial focus was on the distance between the center of mass of the interacting molecules and the lipid components, providing insights into the depth of their insertion within the bilayer. This was closely followed by the analysis of the density distribution using the gmx_density tool, which revealed the spatial localization of OLA and AUR, highlighting their different integration into the lipid environment. Hydrogen bonding was another critical parameter, with the gmx_hbond utility employed to quantify the number of hydrogen bonds formed between the interacting molecules and the lipid headgroups, reflecting the strength and nature of these interactions. Moving forward, the area per lipid was calculated using the MEMBPLUGIN tool [45]. We calculated the area per lipid specifically for the POPC and POPG molecules. This involved measuring the area occupied by each lipid molecule from specific carbon atoms within the glycerol backbone to the terminal carbon atoms of the acyl chains. These calculations aided in understanding the alterations in membrane fluidity and packing density. Specifically, we examined the regions between C2 and C21 (palmitoyl tail) and between C2 and C31 (oleoyl tail) for POPC, and from C1 to C21 and C1 to C31 for POPG. Alongside this, the membrane thickness was assessed by measuring the distance between the phosphorus atoms across opposing monolayers, serving as an indicator of the overall structural integrity and the perturbation induced by the interacting molecules. Another aspect we assessed was the lipid tilt angle, which refers to the orientation of lipid molecules within the membrane. We focused on the phosphorylcholine headgroup in POPC (PN angle) and the glycerol headgroup in POPG [46]. This analysis provided further insights into the structural responses of the membrane under the influence of the inserted molecules. To gain deeper insights into the lateral diffusion of the bilayer, the mean square displacement was measured using gmx_msd, further elucidating the dynamic behavior of the membrane following interaction. The RDF was analyzed to understand the spatial distribution of OLA and AUR relative to the lipid molecules, highlighting their degree of association and the extent of their influence on the membrane structure. Furthermore, the SCD for both POPC and POPG across the *sn*-1 and *sn*-2 positions was employed to assess the ordering of lipid acyl chains, providing a crucial measure of membrane rigidity and dynamic behavior [47]. The various calculated parameters, particularly those using the MEMBPLUGIN tool, such as area per lipid, membrane thickness, lipid tilt, and the SCD, together provide a thorough understanding of how the bilayer behaves when OLA and AUR are introduced. By integrating measurements of distance, density, hydrogen bonding, and other critical factors, we gain a clear picture of how these molecules interact with the lipid membrane.

### Statistical analysis

All data were expressed as the mean ± standard deviation. Each experiment was performed in triplicate. Statistical analyses were conducted using GraphPad Prism software version 8. A one-way analysis of variance (ANOVA) was performed to compare the means between groups, followed by Tukey’s multiple comparison test to identify specific differences between treatment groups. Based on *p*-values, significance was denoted as non-significant (ns) for *p* ≥ 0.05, significant (*) for 0.01 ≤ *p* < 0.05, very significant (**) for 0.001 ≤ *p* < 0.01, and extremely significant (***) for *p* < 0.001.

## Supporting Information

File 1 Additional figures.

## Data Availability

All data that supports the findings of this study is available in the published article and/or the supporting information of this article.
