# Supplementary material for: Mechanistic insights into endosomal escape by sodium oleate-modified liposomes
Source: Beilstein J Nanotechnol. 2024 Dec 30;15:1667–85. doi: 10.3762/bjnano.15.131 (PMC11702302; doi:10.3762/bjnano.15.131)
Supplement: File 1 — Additional figures. [file Beilstein_J_Nanotechnol-15-1667-s001.pdf]

BEILSTEIN JOURNAL OF NANOTECHNOLOGY

Supporting Information

for

Mechanistic insights into endosomal escape by sodium oleate-modified liposomes

Ebrahim Sadaqa, Satrialdi, Fransiska Kurniawan and Diky Mudhakir

Beilstein J. Nanotechnol. **2024,** *15,* 1667–1685. doi:10.3762/bjnano.15.131

Additional figures

License and Terms: This is a supporting information file under the terms of the Creative Commons Attribution License (https://creativecommons.org/licenses/by/4.0). Please note that the reuse, redistribution and reproduction in particular requires that the author(s) and source are credited and that individual graphics may be subject to special legal provisions.
The license is subject to the *Beilstein Journal of Nanotechnology* terms and conditions: (https://www.beilstein-journals.org/bjnano/terms)

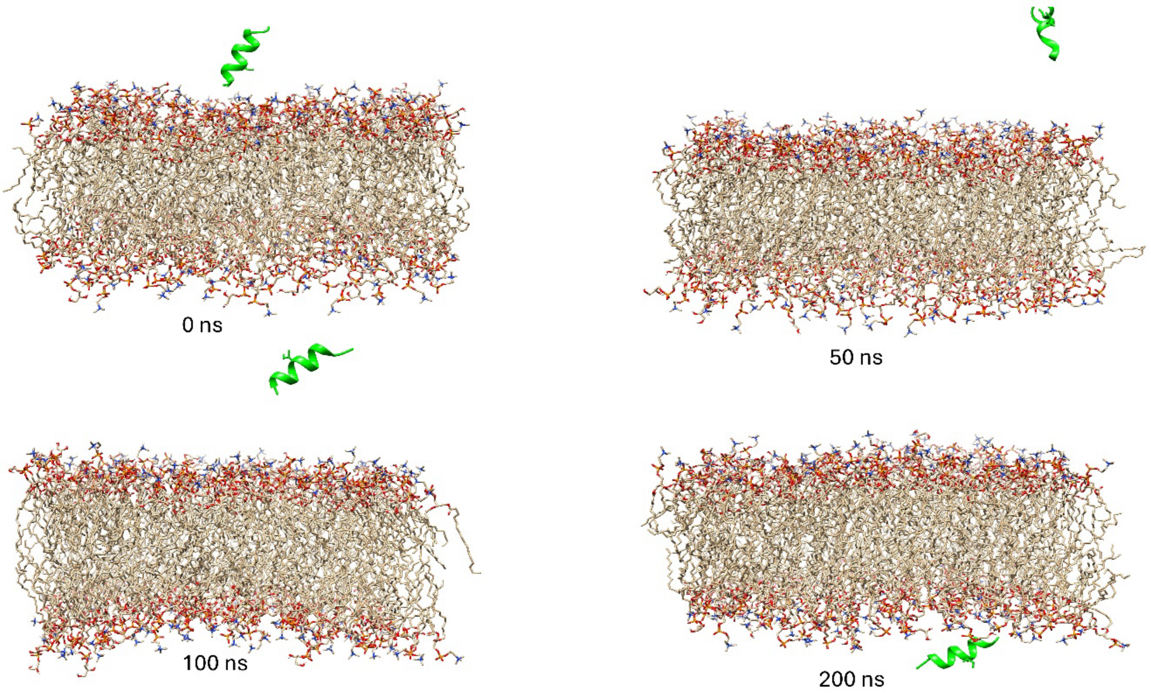

Figure S1: MD simulation of the interaction of non-protonated Aurein 1.2 (AUR) peptide with a lipid bilayer. Snapshots show the position of non-protonated AUR relative to the lipid bilayer after 0, 50, 100, and 200 ns.

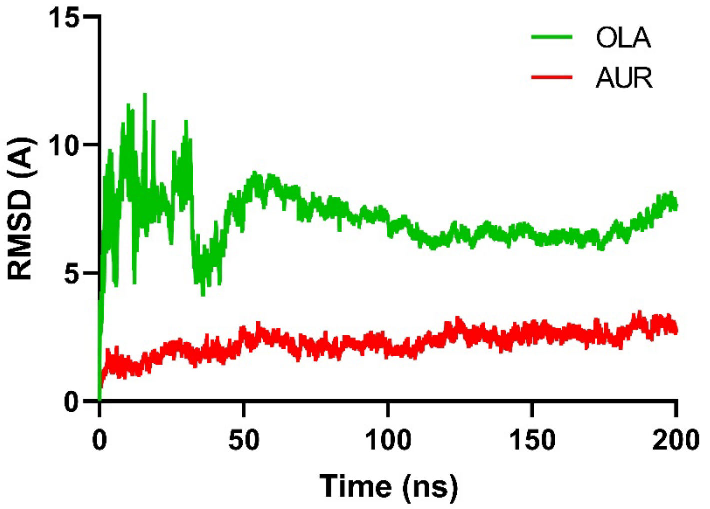

Figure S2: RMSD analysis of oleic acid and Aurein 1.2 in lipid bilayers over 200 ns.

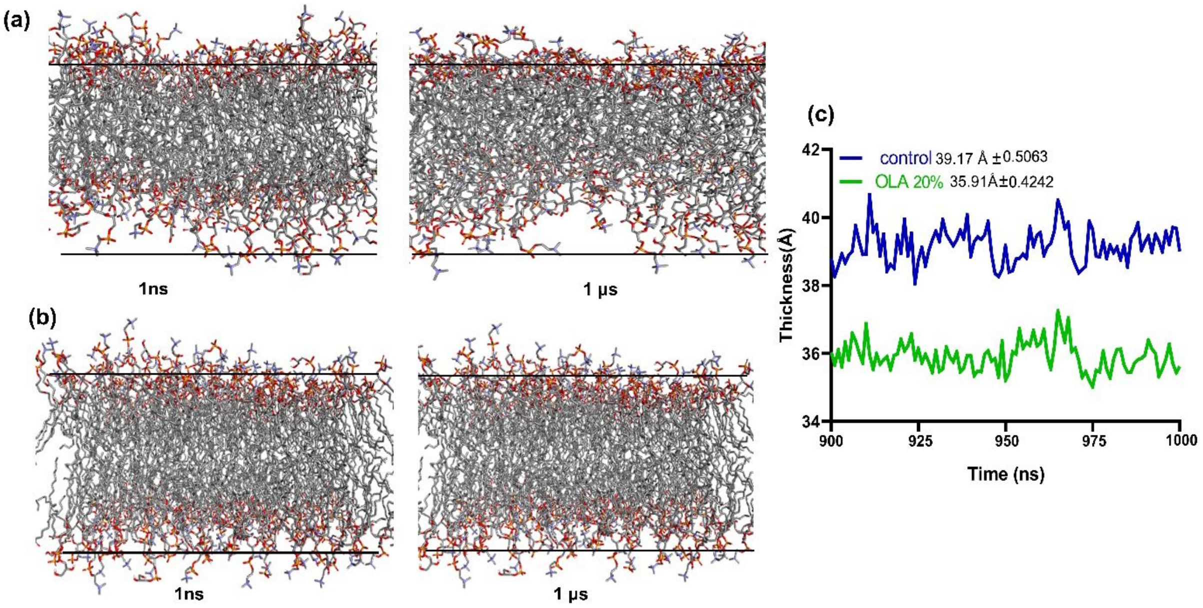

Figure S3: Snapshots of membrane systems at 1 ns and 1 μs for (a) control and (b) 20% oleic acid (OLA). (c) Membrane thickness over the final 100 ns of a 1 μs simulation, showing control (blue) and 20% OLA (green) with respective average thickness values of 39.17 ± 0.51 Å and 35.91 ± 0.42 Å.
